# Provoking the Diagnosis: Coronary Vasospasm Testing in the Setting of Acute Coronary Syndromes

**DOI:** 10.1016/j.jaccas.2026.109456

**Published:** 2026-09-02

**Authors:** Samuel McGrath, Bharat Khialani

**Affiliations:** a Auckland City Hospital, Auckland, New Zealand; b Tan Tock Seng Hospital, National Healthcare Group, Singapore; c Lee Kong Chian School of Medicine, Nanyang Technological University, Singapore

**Keywords:** acetylcholine provocation testing, acute coronary syndrome, coronary vasospasm, MINOCA, ventricular fibrillation

**Figure:**
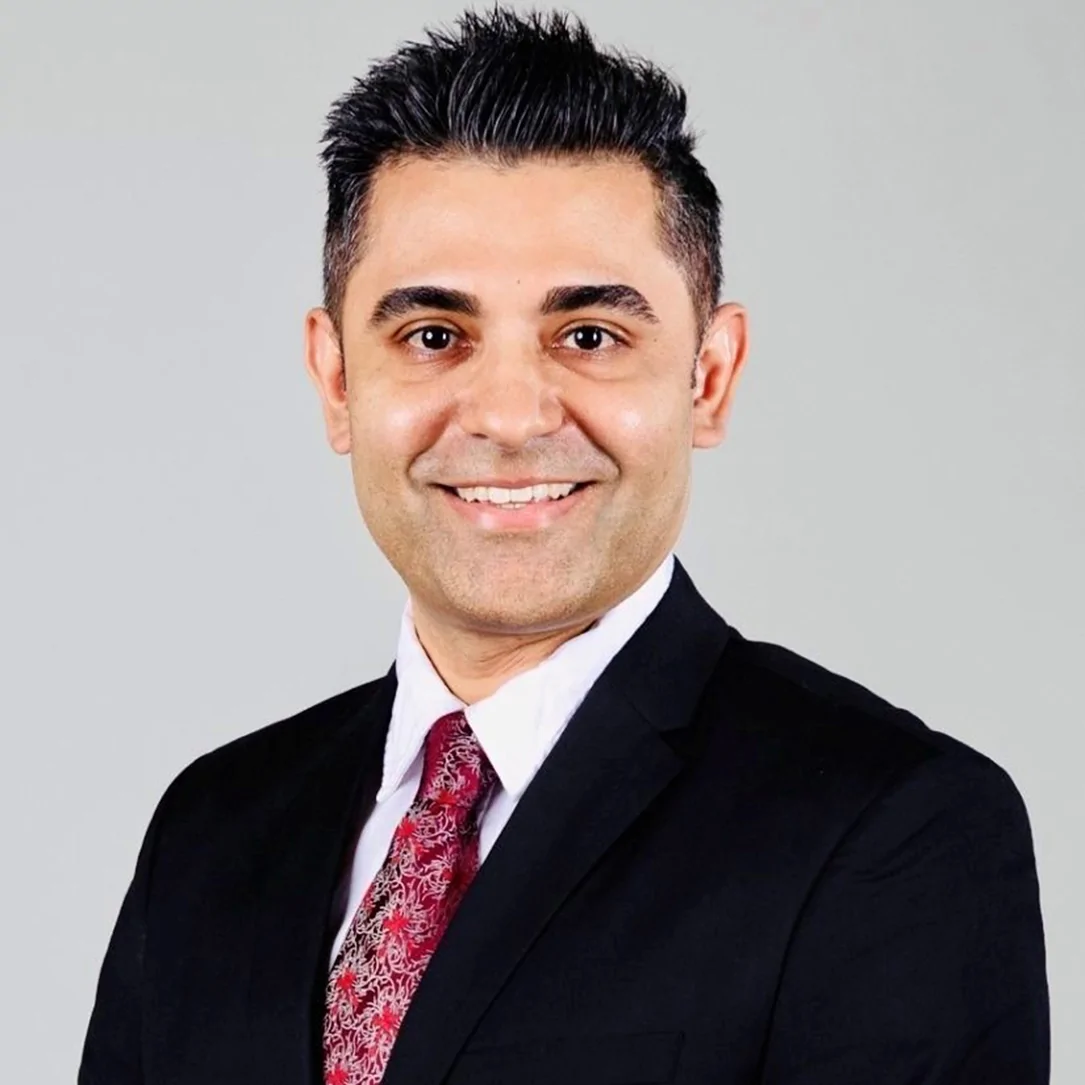

Acute coronary syndromes (ACS) encompass a broad spectrum of presentations, ranging from hemodynamically stable patients with isolated chest pain, to life-threatening emergency presentations such as cardiogenic shock or malignant ventricular arrhythmias. When an ACS presentation is complicated by recurrent or refractory ventricular fibrillation, the diagnostic momentum naturally shifts toward type 1 myocardial infarction caused by atherothrombotic plaque rupture, or scar-mediated arrhythmia, particularly in the setting of known ischemic cardiomyopathy. While this line of reasoning is appropriate for most patients, in a minority, alternative and less common mechanisms such as coronary vasospasm may be responsible. These patients may get labeled as a myocardial infarction with unobstructed coronary arteries (MINOCA), although as the case below illustrates, vasospasm can coexist with hemodynamically significant fixed stenosis, a phenotype that sits at the boundary of both diagnoses.

In this issue of *JACC: Case Reports*, Nishido et al^1^ elegantly illustrate how transient coronary vasomotor dysfunction can produce a clinical phenotype that is every bit as malignant, while leaving no persistent angiographic fingerprints attributable to plaque rupture or thrombosis. What further complicates this case, and broadens its teaching value, is that the patient also had concomitant epicardial coronary artery disease with a functionally significant lesion, raising the question of which substrate drove the arrhythmia. The authors performed a comprehensive physiological and imaging-guided assessment of the lesion, to determine that while the lesion was functionally significant, it was not the root cause of the presentation.

Coronary vasospastic angina (VSA) is defined as reversible and transient vasoconstriction of the coronary vasculature, leading to myocardial ischemia. This phenomenon was first characterized by Prinzmetal et al in 1959.^2^ It is typically thought to cause episodic rest angina, particularly in the mornings and evenings, and can result in transient ST-segment elevation.^3^^,^^4^ This framing, while useful, is incomplete. Severe epicardial spasm can produce profound transmural ischemia, electrical instability, ventricular tachyarrhythmias, cardiac arrest, and presentations that are indistinguishable from conventional ACS at the bedside. The challenge is that the culprit process may be intermittent, nitrate-responsive, and absent at the time of angiography. Without deliberate testing, the diagnosis may be missed, or a presumed diagnosis made on less convincing findings.

This is where vasospasm provocation testing has value. The most established approach for vasoreactivity testing is by intracoronary acetylcholine testing, although ergonovine may be used in some centers.^5^^,^^6^ In selected patients, provocation testing can identify the involved coronary territory, distinguish epicardial from microvascular vasomotor abnormalities, and provide a mechanistic diagnosis that directly changes treatment. A positive test transforms management from generic post-ACS care toward targeted vasodilator therapy, avoidance of triggers, and reconsideration of beta-blockade, which may paradoxically worsen vasospasm by promoting unopposed alpha-adrenergic vasoconstriction in susceptible patients. It can also provide key diagnostic information to support decision-making around device therapy, as evidenced by this case.

Contemporary European Society of Cardiology ACS guidance supports consideration of vasoreactivity testing within the evaluation of MINOCA after stabilization when obstructive culprit disease is not identified.^7^ In the United States, the 2025 American College of Cardiology/American Heart Association ACS guideline is the latest overarching ACS guideline, which predominantly focuses on type 1 myocardial infarction,^8^ and has no specific guidance on vasospasm testing in an ACS setting. The American Heart Association MINOCA scientific statement remains the more specific reference for vasospasm as a potential MINOCA mechanism.^9^ What neither framework adequately addresses is the mixed phenotype illustrated here, in a patient with both hemodynamically significant fixed stenosis and superimposed vasospasm. In this setting, guidelines offer little procedural direction, and clinical judgment must fill the gap. The relevant questions become: Does the fixed lesion independently warrant revascularization on physiological grounds? Could it amplify ischemic vulnerability during spasm by limiting the vasodilatory reserve? And does treating only one substrate leave the patient meaningfully exposed? The present case argues that both mechanisms warrant parallel evaluation and targeted treatment, rather than a sequential or either/or approach. This has implications beyond the individual patient, suggesting that provocation testing should not be withheld simply because obstructive disease is present, provided the clinical picture supports a vasospastic contribution and testing can be performed safely.

While testing in certain ACS presentations is supported by international guidelines, little guidance is given on the timing of the test, especially in a setting as extreme as this case. Timing is central to safety. Acetylcholine can induce bradyarrhythmias, tachyarrhythmias, hemodynamic instability,^10^ as well as the sequelae of myocardial ischemia from inducing coronary spasm. For this reason, provocation testing is not an acute resuscitation tool and should not be performed in unstable patients in whom induced spasm may create unacceptable risk. Rather, it is a diagnostic test to be considered once the patient is stabilized, particularly when angiographic and intracoronary imaging findings do not fully account for the severity of the event. If the clinical picture fits, the diagnostic question should not be limited to “Is there obstructive coronary disease?” but should extend to “Could dynamic coronary obstruction have caused this event?”

The present case is therefore valuable not simply because it demonstrates a rare mechanism of refractory ventricular fibrillation, but because it highlights a recurring blind spot in ACS care. Coronary vasospasm is a dynamic diagnosis in a clinical pathway built largely around fixed anatomy. When the clinical presentation is severe, recurrent, nitrate-responsive, or disproportionate to angiographic disease, clinicians should consider whether provocative testing can reveal the missing mechanism. A proposed pathway is demonstrated in Figure 1. Ultimately, vasospasm testing is not about provoking risk for its own sake, it is about provoking diagnostic clarity. For patients whose first manifestation of coronary vasomotor disease is ventricular fibrillation, cardiac arrest, or ST-segment elevation myocardial infarction, that clarity may be lifesaving.

**Figure 1:**
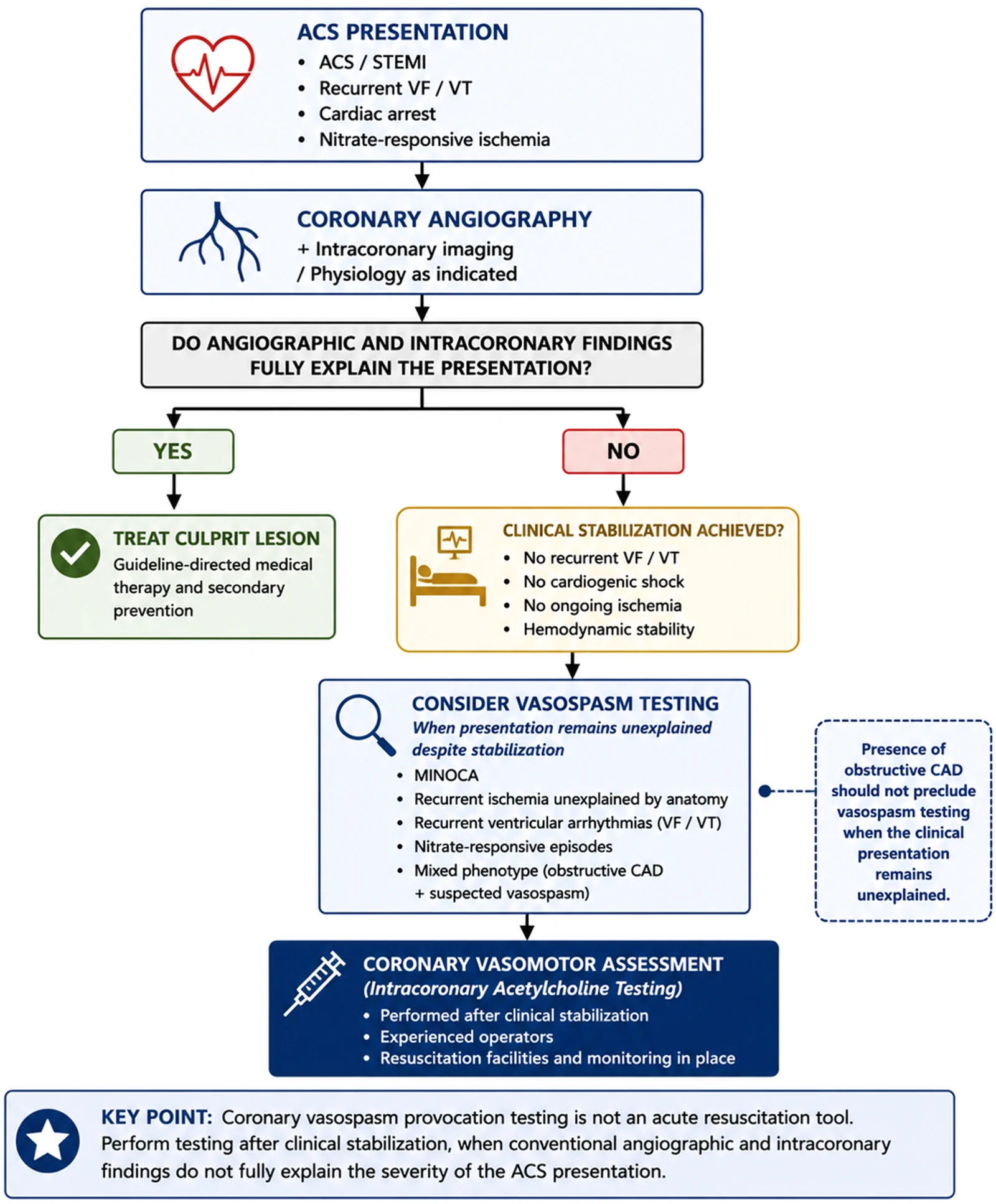
Timing of Coronary Vasospasm Provocation in Acute Coronary Syndromes ACS = acute coronary syndromes; CAD = coronary artery disease; MINOCA = myocardial infarction with unobstructed coronary arteries; STEMI = ST-segment elevation myocardial infarction; VF = ventricular fibrillation; VT = ventricular tachycardia.

## Funding Support and Author Disclosures

The authors have reported that they have no relationships relevant to the contents of this paper to disclose.
